# Health Care Professionals’ Perception of Contraindications for Physical Activity During Cancer Treatment

**DOI:** 10.3389/fonc.2018.00098

**Published:** 2018-04-04

**Authors:** Angeliki Tsiouris, Nadine Ungar, Alexander Haussmann, Monika Sieverding, Karen Steindorf, Joachim Wiskemann

**Affiliations:** ^1^Exercise Oncology, Department of Medical Oncology, National Center for Tumor Diseases (NCT) and Heidelberg University Hospital, Heidelberg, Germany; ^2^Gender Studies and Health Psychology, Institute of Psychology, Heidelberg University, Heidelberg, Germany; ^3^Division of Physical Activity, Prevention and Cancer, National Center for Tumor Diseases (NCT), German Cancer Research Center (DKFZ), Heidelberg, Germany

**Keywords:** medical contraindications, patient safety, physicians, oncology nurses, counseling, physical activity

## Abstract

**Introduction:**

Suggested medical contraindications for physical activity (PA) during cancer therapy might have an influence on PA recommendation behavior of Health Care Professionals (HCP). The purpose of the present study was to examine perceptions of physicians and oncology nurses (ON) toward specific medical conditions as contraindications for PA during cancer treatment.

**Materials and methods:**

A total of 539 physicians and 386 ON were enrolled in this cross-sectional survey. HCP judged 13 medical conditions as to whether they are contraindications for PA during cancer treatment. Answering format was “no contraindication”/“potentially a contraindication”/“yes, a contraindication.”

**Results:**

χ^2^ analyses revealed significant differences between general practitioners, specialized physicians, and ON in their perception of 10 medical conditions. Approximately half of the medical conditions were answered cautiously, showing high numbers on the response option *potentially* (36–72%). Moreover, physicians’ ratings differed significantly depending on their practical experience with particular medical conditions. Those being familiar with a specific medical condition was more permissive to PA during treatment, with effect sizes (Cramer’s *V*) ranging from 0.13 to 0.27.

**Conclusion:**

Results indicate high cautiousness among HCP in judging medical conditions and their impact on PA during cancer treatment. However, group comparisons show that familiarity and clinical experience with potential contraindications facilitate a confident handling of safety issues, which at best leads to higher levels of PA recommendations during cancer treatment.

## Introduction

In recent years, the effectiveness of physical activity (PA) as beneficial and necessary supportive strategy for most cancer survivors has been demonstrated. PA has been shown to improve physical functioning and to positively influence quality of life and mental adjustment to the disease (1–5). Moreover, PA has been associated with a decreased risk of mortality (6–10). Despite the remarkable number of studies confirming the benefits of PA, 60–80% of cancer survivors do not meet the PA guidelines of at least 150 min of moderate-to-vigorous PA/week (11, 12), defined by the American College of Sports Medicine/Centers of Disease Control/American Cancer Society.

Considering the crucial benefits of PA, but also the insufficient activity rates, Health Care Professionals (HCP), like physicians and nurses, play a key role in promoting PA. Having frequent contact during cancer therapy and being the ones cancer survivors have trust in, HCP can encourage cancer survivors to make use of this supportive strategy (13).

Positive attitudes toward PA during cancer treatment were demonstrated on both sides, among HCP (14, 15) as well as among cancer survivors (16). Nevertheless, PA during cancer therapy is not being recommended routinely by HCP (14, 15, 17–20).

Previous research highlighted different types of barriers that might inhibit HCP in giving PA recommendations. Prominent barriers, such as lack of time and reimbursement or missing guidelines, were demonstrated as important structural problems (14). But besides that, studies showed that safety issues can hinder physicians (14, 17, 18) and nurses (21) to recommend PA, as well. Studies assessing the safety concerns of HCP typically used single items that evaluated to what extent physicians and nurses agreed to have safety concerns [e.g., “In my opinion, exercise is safe during treatment” (15)]. This allowed making a statement about how concerned they are in general about safety of PA during cancer treatment, but it did not provide further insights into which medical contraindications and safety issues exactly account for their concerns.

In fact, there is a broad spectrum of conditions in which PA is contraindicated, e.g., extreme anemia or fatigue, fever, or an acute infection (22). However, there is only a limited amount of empirical data on which to refer when evaluating specific contraindications and safety issues (23), so that physicians’ and nurses’ judgments on safety issues are assumed to be influenced by their previous clinical experience. A profound understanding of the attitudes and uncertainties of HCP toward medical conditions as contraindications might help to detect where the greatest need for further research and education lies. More precisely, it would help to develop measures that are customized to the needs of the particular HCP subgroups [physicians of different medical specialties, oncology nurses (ON)] as well as to identify specific medical conditions where safety concerns are especially high.

Therefore, the aim of the present study was (1) to investigate to what extent specific medical conditions and safety issues are perceived as having an influence on the feasibility of PA during cancer therapy and (2) to explore whether physicians of different disciplines differ in their perception of the influence of medical conditions on PA. Thereby, we assumed that being familiar with a particular medical condition, which often comes along with the medical specialization of the physician, helps physicians to assess the relevance of that specific condition appropriately and confidently. Finally, it should be exploratory investigated whether the general perception of medical contraindications for PA is associated with professional variables and PA recommendation behavior.

## Materials and Methods

### Sample and Procedures

This cross-sectional study was conducted as a cooperation project (Momentum Project) between the National Center of Tumor Diseases, the Heidelberg University Hospital, the German Cancer Research Center, and the Psychological Institute of Heidelberg University. The study received ethical approval by the ethics commission of the Faculty of Behavioral and Cultural Studies of Heidelberg University.

The recruitment took place from February 2016 until July 2016. Eligible participants consisted of general practitioners (GP), gynecologists, gastroenterologists, urologists, surgeons, medical oncologists, radiation oncologists, and ON, provided that they had regular contact to survivors with breast, prostate, or colon cancer. This group was targeted for two reasons. Firstly, because breast, prostate, and colon cancer represent prevailing cancer entities; secondly, because benefits of PA during therapy for these cancer sites are comprehensively investigated so far (24). Participants could complete either the paper pencil or the online version of the questionnaire. Online participation was enabled in order to increase the spread of the questionnaire and thus the number of participants.

A sample of 2,203 potential participants (1,543 physicians and 660 nurses) was randomly drawn on basis of compulsory listings of resident physicians and hospitals in Germany. The number of potential participants and hospitals that were contacted in each of the 16 German states was selected proportionately to its population. Additionally, 422 questionnaires were handed out to HCP at different national medical congresses in 2016. Each potential participant received a questionnaire package that consisted of detailed study information, informed consent, the questionnaire, and a stamped return envelope. Resident physicians who did not respond to the mailed questionnaire package were reminded *via* email with reference to the option of participating online.

The online questionnaire was congruent to the paper pencil version. Participants agreed to the informed consent online. The online questionnaire was mainly distributed through promoting the study in different magazines, newsletters, or homepages for physicians and nursing staff, such as monthly journals of regional medical associations or medical specialists associations. Thus, the online questionnaire was also accessible to volunteers whose interest was gained by the various promoting strategies, but who were not directly contacted by the project team. When promoting the study, we did not particularly focus on PA, but in general on supportive strategies during cancer therapy. This was done in order to not attract only those, who had a great interest in PA and, thus, to reduce selection bias (14, 15). Each participant received 25€ as incentive for completing the questionnaire. Additionally, personalized cover letters and assurance of confidentiality were used in order to increase response rates.

### Measures

#### Medical Conditions as Contraindications for PA

Participants were presented a list of 13 medical conditions: port, ostomy, acute infection, ongoing radiation, incomplete wound-healing, leukopenia, palliative situation, ongoing chemotherapy, cachexia, increasing pain during exercise, no medical exercise preparticipation check available, platelet count of 50,000/μl, and vertebral bone metastases. For every medical condition, participants were asked: “*Does this represent a medical contraindication for PA in cancer survivors?*” Response options were *no*/*potentially*/*yes*. As there is no standardized scale for assessing the perception of contraindications, this scale was developed and pre-tested in two steps. First, a qualitative pre-test was conducted. Therefore, a group of *N* = 30 HCP [10 of each group: GP, specialized physicians (SP), and ON] were interviewed and asked about safety concerns and other inhibiting factors for PA (recommendation). These interviews (mean duration = 29 min, SD = 8.5 min) were carried out in person or by phone (25). Based on results of the qualitative pre-test, the scale assessing medical contraindications was evaluated in a quantitative pre-test within a sample of *N* = 88 HCP. Following both pre-tests, we considered the selected items as appropriate for the purpose of our study. The selection of these particular medical conditions was primarily based on safety issues described in the ACSM Roundtable on Exercise Guidelines for cancer survivors (22). Additionally, we considered medical conditions noted in National Comprehensive Cancer Network guidelines as well as frequently mentioned exclusion criteria in exercise intervention studies with cancer survivors.

#### PA Recommendation Practice

One item assessed PA recommendation practice of HCP. This item asked participants to state how often they provided PA recommendations to cancer survivors in the past 3 months. Participants chose between five response options: “*I advise against being physically active*,” “*never/rarely (<10%)*,” “*sometimes (10–50%)*,” “*often (50–90%)*,” “*on most/every visit (>90%)*.

#### Demographic and Professional Information

Demographic and professional information included age, sex, clinical specialization, years of practice, number of cancer survivors seen per month, primarily treated tumor types, and primarily administered treatment types. Finally, participants were asked to estimate the percentage of cancer survivors they treat with either curative or palliative intent. Both values (curative and palliative intent) should sum up to 100%.

In consideration of selection bias, the questionnaire was designed in a way that it asked for general attitudes toward the broader spectrum of supportive strategies at the beginning, and then limited the scope on PA later on. In order to create a common understanding of PA, a short description of the type of PA relevant for the study was provided. HCP were asked to think of intended PA, which is at least perceived as slightly exhausting, such as Nordic walking, swimming, strength training, or jogging. Finally, the definition included that light PA, such as slow walking, was not meant as referred type of PA.

### Statistical Analyses

Analyses of data were conducted using SPSS (version 22). A significant *p*-value was set at α = 0.05. First, descriptive analyses were used for demographic and professional information. Further descriptive analyses revealed HCP perception of contraindications. Significant group differences between GP, SP, and ON in the perception of medical conditions were determined using χ*^2^*-analysis. Strength of association in this comparison was evaluated using contingency coefficient *C*. Contingency coefficients (*C*) of 0.10, 0.30, and 0.50 represent small, medium, and large effects (26). On the basis of calculated effect sizes, medical conditions were categorized in (1) judged with agreement, (2) judged with low disagreement, or (3) judged with medium disagreement across the HCP groups. Finally, if the χ*^2^*-analysis revealed significant differences between the three groups, pairwise comparisons were conducted using Fisher’s exact test.

In order to find out, if familiarity with a medical condition influences its perceived impact on PA, we conducted further group comparisons. Only data from physicians were included in this analysis. Medical conditions that could primarily be assigned to the task area of a specific medical specialization were additionally analyzed by dividing physicians into two groups: experts vs. others. Thus, for example, oncologists were assumed to be experts in assessing the impact of an ongoing chemotherapy on PA promotion, and were compared with a group that included the remaining physicians. Analogically, comparison groups were formed for the safety issues *platelet count of 50,000/μl, cachexia, leukopenia*, and *ongoing radiation*, whereby the group formation of experts vs. others varied on a case-by-case basis. Medical conditions that could not be clearly identified as subjects of a particular expert group were not assigned to experts vs. others. Classification was undertaken by Joachim Wiskemann and Angeliki Tsiouris with regard to the clinical and practical experience of the various medical specialties with a particular medical condition. Group differences between experts and others were calculated using χ*^2^*. In this analysis, Cramer’s V (*V)* was used as indicator for strength of association. Effect sizes of 0.10, 0.30, and 0.50 were considered as small, medium, and large effects (26).

For comparing individuals in their overall perception of medical contraindications, an individual aggregated value was calculated. For that, all ratings given by one participant were put in ratio by weighting the number of *no*-, *potentially*-, and *yes*-answers with a factor [(sum *no*-answers*0 + sum *potentially* answers*0.5 + sum *yes*-answers*1)/number of valid answers]. By means of this aggregated contraindication score, subgroups were compared in their overall tendency to rate the influence of medical conditions rather permissively or rather strictly, using analyses of variance and *t*-tests for independent samples.

## Results

Of the 2,625 mailed questionnaires, eight questionnaires were returned as undeliverable, resulting in a total of 2,617 eligible participants contacted *via* mail. Response rate for the paper pencil version was 19.3% for physicians (358/1.857) and 30.8% for ON (233/760), in total 22.7%. Furthermore, 365 participants (194 physicians, 171 ON) completed the online questionnaire. Response rate for the online version cannot be calculated, since the online questionnaire was freely available to volunteers, and not reserved to a selected sample. Online participants who only answered on demographic items (*N* = 56) were excluded from analysis. The same was done for online participants who participated repeatedly (*N* = 15). Participants who did not answer any of the 13 medical conditions were excluded from analyses (13 physicians/18 ON), resulting in a total of 925 responders to the study.

Demographic and professional information is displayed in Table 1. In brief, the sample consisted of 539 physicians (age M = 46.1 ± 11.4; 53% male) and 386 ON (age M = 39.5 ± 10.4; 18% male).

**Table 1:** Descriptive statistics of demographic and professional information.

	Physicians (*N* = 539)	Oncology nurses (*N* = 386)
	Frequency (%) or M ± SD	Frequency (%) or M ± SD
**Demographic and professional information**		
Age	46.1 ± 11.4	39.5 ± 10.4
<45 years	228 (42%)	247 (64%)
≥45 years	311 (58%)	139 (36%)
Sex		
Male	283 (53%)	68 (18%)
Female	255 (47%)	311 (82%)
Medical specialization		
General practitioners	159 (30%)	–
Medical oncologists	63(12%)	–
Radiation oncologists	62 (11%)	–
Gastroenterologists	46 (9%)	–
Urologists	65 (12%)	–
Gynecologists	76 (14%)	–
Surgeons	50 (9%)	–
Other medical specialties	18 (3%)	–
Number of years in practice	17.5 ± 11.0	19.3 ± 10.5
<15 years	216 (40%)	153 (40%)
≥15 years	323 (60%)	233 (60%)
**Patients- and treatment-related characteristics**		
Number of patients seen/month	60.1 ± 79.7	82.4 ± 107.0
General practitioners	22.3 ± 27.1	–
Medical specialists	75.7 ± 88.6	–
Primarily treated tumor types^a^		
Breast	349 (65%)	209 (54%)
Prostate	327 (61%)	199 (52%)
Colorectal	371 (69%)	246 (64%)
Other	235 (44%)	194 (50%)
Treatment types^a^		
Chemotherapy	415 (77%)	305 (79%)
Radiation	321 (60%)	226 (59%)
Surgery	410 (76%)	213 (55%)
Aftercare	399 (74%)	151 (39%)
Others	95 (18%)	63 (16%)
PA recommendation practice		
Advising against PA	2 (0%)	1 (0%)
Never/rarely recommend PA	10 (2%)	27 (7%)
Sometimes recommend PA (10–50%)	73 (14%)	76 (20%)
Often recommend PA (50–90%)	190 (35%)	156 (40%)
Recommend PA on most/every visit (>90%)	263 (49%)	125 (32%)

*^a^Multiple responses are possible*.

*Numbers may not sum up to N = 539 or N = 386 for physicians or oncology nurses due to missing values*.

*M, mean; PA, physical activity*.

Table 2 displays descriptive results of the perception of medical conditions and group differences for GP, SP, and ON.

**Table 2:** Response frequencies (in percent) of Health Care Professionals on medical conditions.

“*Do the below defined medical conditions represent a contraindication for physical activity in cancer patients?*”	General practitioners (GP)			Specialized physicians (SP)			Oncology nurses (ON)			Statistical tests of group differences			
		
	*N* = 158–159			*N* = 376–380			*N* = 380–386						
			
	No (%)	Pot. (%)	Yes (%)	No (%)	Pot. (%)	Yes (%)	No (%)	Pot. (%)	Yes (%)	χ^2^	*p*-Value	Effect size (*C*)^a^	Sign. group differences between^b^
**Agreement between groups and certainty in how to rate medical condition**													
Port	93	4	3	96	3	1	94	4	2	4.74	0.32	0.07	–
Ostomy	88	10	2	90	9	1	86	12	2	5.31	0.26	0.08	–
Acute infection	1	19	80	2	24	74	4	22	74	7.73	0.10	0.09	–

**Low disagreement between groups in how to rate medical condition**													
Ongoing radiation	62	32	6	73	24	3	69	25	6	9.52	0.049	0.10	GP–SP
Incomplete wound-healing	17	72	11	11	70	19	14	61	25	15.88	0.003	0.13	GP–SP, GP–ON
Leukopenia	31	55	14	33	48	19	25	46	29	21.46	<0.001	0.15	GP–ON, SP–ON
Palliative situation	69	25	6	78	21	1	64	28	8	28.57	<0.001	0.17	SP–ON
Ongoing chemotherapy	46	49	5	65	27	8	67	27	6	29.15	<0.001	0.18	GP–SP, GP–ON
Cachexia	28	60	12	48	44	8	56	38	6	36.81	<0.001	0.20	GP–SP, GP–ON
Increasing pain during exercise	11	61	28	8	60	32	4	47	49	38.69	<0.001	0.20	GP–ON, SP–ON

**Medium disagreement between groups in how to rate medical condition**													
No medical exercise preparticipation check	88	12	0	84	14	2	59	36	5	82.55	<0.001	0.29	GP–ON, SP–ON
Platelet count of 50,000/μl	45	43	12	52	36	12	20	52	28	97.41	<0.001	0.31	GP–ON, SP–ON
Vertebral bone metastases	16	72	12	23	67	10	4	55	41	141.65	<0.001	0.37	GP–ON, SP–ON

*^a^Contingency coefficients (*C*) of 0.10, 0.30, and 0.50 represent small, medium, and large degrees of association [according to Cohen (26)]*.

*^b^Based on Fisher’s Exact test. All group differences (*N* = 18) are significant at *p* < 0.05; *N* = 15 group differences are significant at *p* < 0.001*.

*Pot., potentially*.

### Similarities and Differences in the Perception of Medical Conditions as Potential Contraindications Between HCP Groups (GP, SP, ON)

There were no significant differences, and thus a general agreement, between the subgroups that having a *port* and having an *ostomy* do not represent a contraindication to PA (M_no_ = 94 and 88%), while *acute infection* was clearly rated as a contraindication (M_yes_ = 76%). These were the only cases, where the three HCP subgroups answered similarly, without significant differences.

The remaining medical conditions revealed significant differences and display low to medium disagreement between groups. ON tended to have a higher number of *yes*-answers and thus evaluated more medical conditions as contraindications, than physicians did (*incomplete wound-healing, leukopenia, increasing pain during exercise, platelet count of 50,000/μl*, and *vertebral bone metastases*). However, GP and SP disagreed in some medical conditions as well. SP showed higher proportions of *no*-answers and thus more tolerance for PA despite of *ongoing radiation, ongoing chemotherapy*, and *cachexia*, compared with GP.

Across the three subgroups, *leukopenia, increasing pain during exercise, vertebral bone metastases*, and *incomplete wound-healing* turned out to be perceived most cautiously, as measured by the high proportion of *potentially* answers. The medical conditions *cachexia* and *platelet count of 50,000/μl* revealed lower, but still remarkable cautiousness.

### Comparing Perceptions of Expert Physicians With Other Physicians

**Figure 1:**
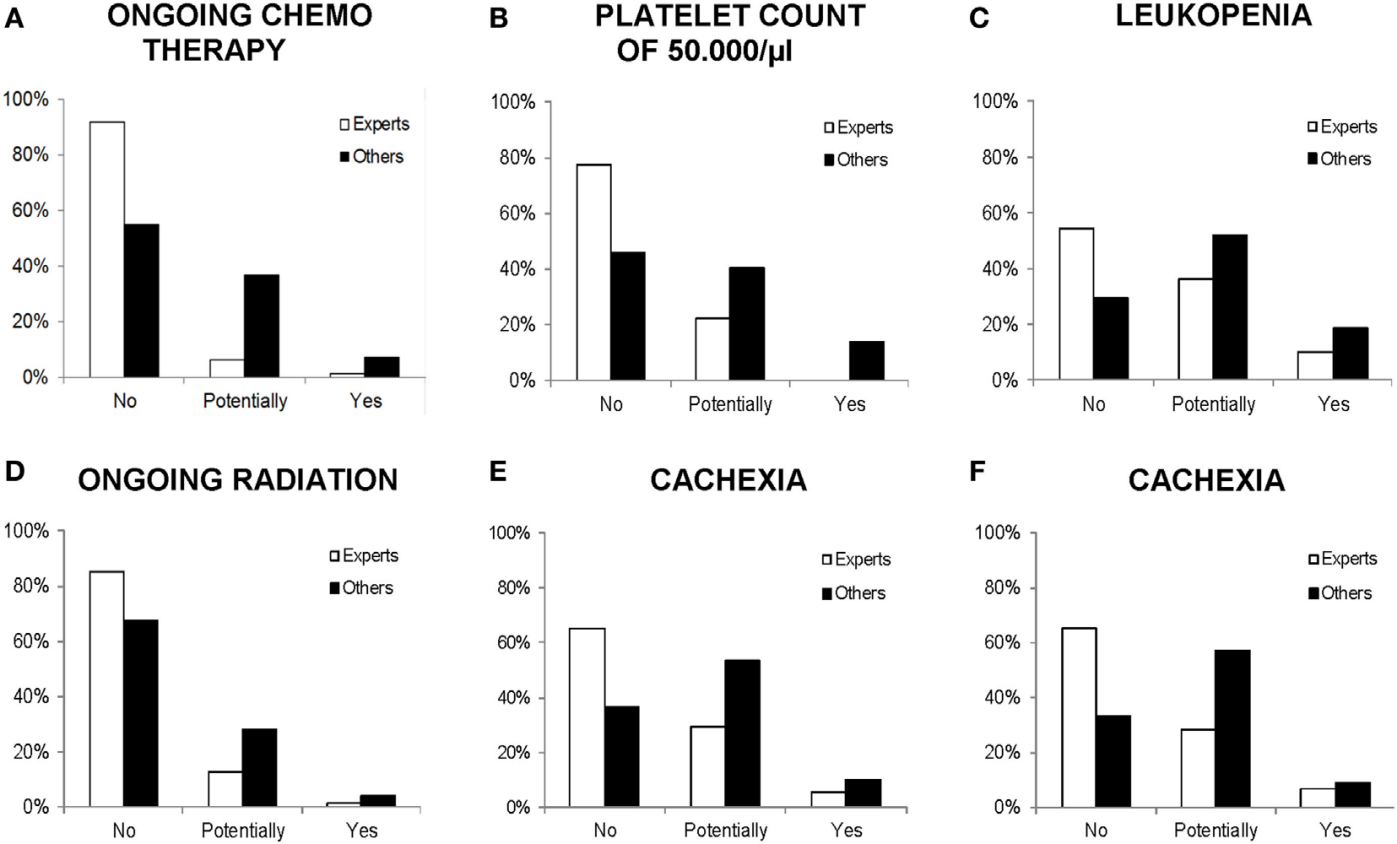
Comparison of physicians’ response frequencies (in percent) on particular medical conditions with the grouping factor experts vs. others. All differences are significant at *p* < 0.05. **(A–C)** Experts: medical oncologists, others: other specialties. **(D)** Experts: radiation oncologists, others: other specialties. **(E)** Experts: medical oncologists and gastroenterologists, others: other specialties. **(F)** Experts: physicians treating with palliative intent (>60% palliative patients per month), others: physicians treating with curative intent (>60% curative patients per month).

Group differences between expert vs. other physicians are displayed in Figure 1. Medical specialists who can be regarded as experts for a certain medical condition responded less strictly on it. Hence, medical oncologists were more permissive toward the influence of *ongoing chemotherapy* [χ*^2^*(2) = 30.46, *p* < 0.001, *V* = 0.23], *platelet count of 50,000/μl* [χ*^2^*(2) = 24.83, *p* < 0.001, *V* = 0.22], and *leukopenia* [χ*^2^*(2) = 15.23, *p* < 0.001, *V* = 0.17] on PA, compared with other physicians. Analogically, radiation oncologists were significantly more permissive to PA during *ongoing radiation*, compared with other physicians [χ*^2^*(2) = 8.40, *p* = 0.015, *V* = 0.13]. Significant differences in the perception of *cachexia* due to expert knowledge were shown in both grouping factors: medical oncologists and gastroenterologists as experts vs. others [χ*^2^*(2) = 29.10, *p* < 0.001, *V* = 0.23] and physicians treating with palliative intent vs. physicians treating with curative intent (others) [χ*^2^*(2) = 30.18, *p* < 0.001, *V* = 0.27].

Differences in the overall perception of medical conditions (aggregated contraindication score) based on further professional characteristics of HCP are displayed in Table 3. Differences in the perception based on sex and age were calculated separately for physicians and ON. Within these groups, there are no significant differences between male and female participants. However, the classification of age (under or over 45 years) revealed significant differences in both groups, physicians [*t*(533.87) = −3.57, *p* = < 0.001, *d* = 0.29] and ON [*t*(384) = 2.03, *p* = 0.044, *d* = 0.19]. HCP differed significantly in their overall perception of medical conditions, depending on their professional background [*F*(7,899) = 11.43, *p* < 0.001, η*^2^* = 0.08]. Medical oncologists had the lowest, while ON had the highest aggregated contraindication score (*M* = 0.24 vs. 0.39). Moreover, HCP who stated a rather palliative treatment intent showed lower overall scores, which stand for a less strict attitude [*t*(665) = 3.42, *p* = 0.001, *d* = 0.27]. Furthermore, a less rigorous attitude toward medical conditions was also shown in HCP who recommend PA on at least or more than 90% of their visits [*t*(897.85) = 5.82, *p* < 0.001, *d* = 0.41].

**Table 3:** Differences in aggregated contraindication scores^a^ (M/SD) based on demographic and professional variables.

	M (SD)	*F*	*t*	*p*-Value	Effect size^b^
**Physicians**					
Sex					
Male (*N* = 283)	0.32 (0.14)		1.83	0.067	0.15
Female (*N* = 255)	0.34 (0.13)				
Age					
<45 years (*N* = 228)	0.30 (0.12)		−3.57	<0.001	0.29
≥45 years (*N* = 311)	0.34 (0.15)				

**Oncology nurses**					
Sex					
Male (*N* = 68)	0.37 (0.16)		1.18	0.239	0.13
Female (*N* = 311)	0.39 (0.15)				
Age					
<45 years (*N* = 247)	0.40 (0.15)		2.03	0.044	0.19
≥45 years (*N* = 139)	0.37 (0.16)				

**Physicians and oncology nurses**					
Professional background					
General practitioners	0.34 (0.13)	11.43	–	<0.001	0.08
Medical oncologists	0.24 (0.09)				
Radiation oncologists	0.34 (0.13)				
Gastroenterologists	0.30 (0.14)				
Urologists	0.36 (0.13)				
Gynecologists	0.36 (0.15)				
Surgeons	0.31 (0.16)				
Oncology nurses	0.39 (0.15)				
HCP treating with curative intent (*N* = 494)	0.36 (0.15)	–	3.42	0.001	0.27
HCP treating with palliative intent (*N* = 173)^c^	0.32 (0.14)				
HCP who recommend PA on <90% of their visits (*N* = 535)	0.38 (0.16)	–	5.82	<0.001	0.41
HCP who recommend PA on ≥90% of their visits (*N* = 388)	0.32 (0.13)				

^a^The aggregated contraindication score is a result of the equation [(sum no-answers*0 + sum potentially answers*0.5 + sum yes-answers*1)/number of valid answers] calculated for each participant and then averaged for the different subgroups. This score can adopt values between 0 and 1. Higher values indicate a more cautious perception of medical conditions as contraindications.

*^b^Effect sizes: partial η^2^ for the ANOVA, d for t-tests*.

*^c^HCP treating with curative intent: >60% curative patients; HCP treating with palliative intent: >60% palliative patients*.

*M, mean; HCP, health care professionals; PA, physical activity*.

## Discussion

The present study sought to clarify HCPs’ perception of potential medical contraindications for PA during cancer therapy. For this purpose, the study provided several detailed and essential insights.

While there were some few medical conditions that were consistently characterized as no or as clear contraindications, for many other conditions there were high rates of *potentially* answers and higher disagreement across HCP groups. Except for having an *ostomy* and having a *port*, which were rated as not hindering for PA recommendations, and an *acute infection*, which was assessed as a clear contraindication, ratings were ambiguous and inconsistent between and within the subgroups. Prominent and frequently discussed safety issues like *platelet count of 50,000/μl, cachexia, vertebral bone metastases, ongoing chemotherapy*, or *radiation* were answered with caution.

In this context, it is necessary to mention that caution in prescribing PA to cancer survivors is reasonable and helps preventing from exercise-induced events. A cautious approach, however, can become a problem if it leads HCP to abstain from recommending PA, although the condition of a cancer survivor would allow being physically active. When interpreting the results, it seems justified to consider that caution might also reflect uncertainty among HCP in how to evaluate medical conditions when prescribing PA.

For several reasons, the cautious or uncertain stance of HCP is plausible. So far, there is only little, if any, structured, empirical evidence, which clarifies the relevance of specific medical contraindications for PA in cancer survivors (23). As contraindications and safety issues can hardly be main research subjects, knowledge about potential medical contraindications is mostly extracted from exercise intervention studies with cancer survivors that report exclusion criteria and adverse effects. Reported exclusion criteria and adverse effects might give a hint whether a medical situation can be assessed as a risk factor that requires particular precautions. However, it is important to note that most studies use strict exclusion criteria and focus on quite “healthy” cancer populations [e.g., early stage breast or prostate cancer survivors (27)] and only a few are dealing with advanced or higher risk populations (28). In turn, studies with strict exclusion criteria and with “healthy” cancer populations, will scarcely contribute to increase knowledge about medical contraindications for PA among cancer survivors. Referring to this, it is essential to underline that several studies got to the promising conclusion that adverse effects due to exercising are infrequent (22) and the risk of exercise-induced events is low (29), concluding that PA is safe for people with cancer (30).

Another important finding arose from the comparison of the HCP subgroups and especially from the expert-comparison. Significant subgroup-differences indicate that familiarity and practical experience with specific medical conditions enhance a confident handling of these conditions, when it comes to a PA consideration. Particularly worthy of emphasis are the judgments of medical oncologists. Being those who are most familiar with low-platelet count, leukopenia, ongoing chemotherapy, and cachexia, due to their day-to-day work, medical oncologists had the most allowing perception of these medical conditions in regard to PA. Moreover, the judgments of experts support the assumption, that uncertain medical conditions should not be seen as reason to not recommend PA at all, but as a reason to tailor a PA recommendation to the preexisting medical condition.

In consequence, we assume confidence in judging medical conditions to result in an increased level of modified PA promotion, which considers the individual condition of every cancer survivor adequately. Thus, cancer survivors might be encouraged to adopt a PA behavior that is feasible and takes into account their individual needs (24). Taking up this approach could be an important step toward a comprehensive exercise therapy provision, which reaches a large majority of cancer survivors.

Overall, the results reflect the need for further clinical and empirical research in order to encounter the concerns raised in this study. Therefore, further Phase 1 trials can help augmenting the existing knowledge of PA safety and medical contraindications during cancer therapy. Additionally, a roundtable on medical contraindications and safety issues, which brings together research and clinical experience, could be a possible initiative. Compiled knowledge could be aggregated in official guidelines in order to reach as many HCP as possible. Furthermore, professional exchanges of HCPs with different medical backgrounds are worth considering. In this context, HCP with little experience in judging the influence of specific medical conditions on PA can benefit from their more experienced colleagues.

Although the present study provides important information about how HCP perceive the influence of medical conditions as contraindications for PA during cancer therapy, there are several limitations that need to be considered when interpreting the results. One main limitation is the way the perceptions of medical conditions were assessed. In order to gain more precise ratings, it would be preferable to define some medical conditions in greater detail (e.g., *incomplete wound-healing* with additionally mentioning the affected body region). It cannot be ruled out that HCP judgments of medical conditions could have been different, if they were provided with more specific information on the particular conditions. Another limitation refers to the answering format (*no/potentially/yes*). The item was conceptualized in a way that it allowed participants to express indecisiveness by answering *potentially*. As already mentioned, we can interpret answers on *potentially* as uncertainty, but we have to consider that this could also be seen as an intended and certain answer of HCP, meaning that they need more detailed information for a decision or that they are convinced that decision would differ on an individual patient level. This limitation might be countered by using a gravity scale, on which participants rate the relevance of medical conditions for PA. Finally, one important limitation refers to the isolated consideration of each medical condition. Medical conditions normally do not occur in an isolated state, but are rather associated with each other. In the present study, these interactions between medical conditions remain unconsidered.

A major strength of this study, which is the first one to investigate attitudes of HCP in the area of PA during cancer treatment in a German population, is the broad data base. The wide professional diversity of physicians revealed insights from different perspectives. Moreover, the large variety of recruiting strategies sought to obtain a random and highly representative sample. Following up on this, the response bias, attracting especially HCP who are interested in PA, was countered by disguising the main intention of the study.

## Conclusion

In conclusion, the results suggest cautiousness in judging particular medical conditions as contraindications for PA. For some medical conditions, this cautiousness might also reflect uncertainty among HCP. As uncertainty could impact PA promotion behavior, the present findings underline the need for further clinical and empirical research. Future exercise intervention studies and Phase I Trials including higher risk cancer survivors and Roundtable Consensus meetings can provide remedy to the uncertainty of HCP. However, results also indicate that familiarity and broad clinical experience with particular medical conditions facilitate a safe and confident handling of safety issues.

Taken this into account, empirical and clinical progress on this topic will presumably help HCP to give safe and frequent PA recommendations that respect the individual health status of each cancer survivor.

## Ethics Statement

The study received ethical approval by the ethics commission of the Faculty of Behavioral and Cultural Studies of Heidelberg University. All procedures performed were in accordance with the 1964 Helsinki declaration and its later amendments or comparable ethical standards. Informed consent was obtained from all individual participants included in the study.

## Author Contributions

All authors contributed to the data collection, manuscript writing, and final approval of the manuscript. NU, AH, MS, KS, and JW contributed to the conception of the study. AT and JW contributed to the data analysis and interpretation.

## Conflict of Interest Statement

The authors declare that the research was conducted in the absence of any commercial or financial relationships that could be construed as a potential conflict of interest.
